# The early transcriptional and post-transcriptional responses to fluconazole in sensitive and resistant *Candida albicans*

**DOI:** 10.1038/s41598-024-80435-w

**Published:** 2024-11-22

**Authors:** Irene Stevens, Fitz Gerald Silao, Susanne Huch, Honglian Liu, Kicki Ryman, Adriana Carvajal-Jimenez, Per O. Ljungdahl, Vicent Pelechano

**Affiliations:** 1 Science for Life Laboratory (SciLifeLab), Department of Microbiology, Tumor and Cell Biology, Karolinska Institutet, Solna, Sweden; 2 SciLifeLab, Department of Molecular Biosciences, The Wenner-Gren Institute, Stockholm University, Stockholm, Sweden

**Keywords:** Fungal genomics, RNA decay, Transcriptomics, RNA sequencing, Pathogens

## Abstract

*Candida albicans* is a leading cause of fungal infections in immunocompromised patients. Management of candidemia relies on a few antifungal agents, with fluconazole being first line therapy. The emergence of fluconazole-resistant strains highlights the pressing need to improve our molecular understanding of the drug response mechanisms. By sequencing the 5’P mRNA degradation intermediates, we establish that co-translational mRNA decay occurs in *C. albicans* and characterize how in vivo 5´-3´ exonuclease degradation trails the last translating ribosome. Thus, the study of the 5’ Phosphorylated mRNA degradome (5PSeq) offers a simple and affordable way to measure ribosome dynamics and identify codon specific ribosome stalls in response to drugs and amino acid deprivation. Building upon this, we combine RNA-Seq and 5PSeq to study the early response of sensitive and resistant *C. albicans* isolates to fluconazole. Our results highlight that transcriptional responses, rather than changes in ribosome dynamics, are the main driver of *Candida* resistance to fluconazole.

## Introduction

*Candida albicans* is a biofilm-forming commensal associated with substantial healthcare costs and a high global burden of infections^1,2^. While fluconazole is a widely used and well tolerated treatment for candida infections, its repeated use or long-term prophylaxis has led to the emergence of resistant strains, creating a threat to public health^3^.

Understanding gene expression is crucial for deciphering how cells adapt to external perturbations, such as stress or drugs. Most studies investigating gene expression focus on mRNA abundances (transcription levels). Previous studies have examined fluconazole-induced transcriptional responses in candida, as early as 15 min^4^. This has revealed that fluconazole exposure rewires gene expression pathways related to cellular stress aiding in survival . Several other studies have identified stress pathways as potential drug targets for resistant infections^5,6^, including the Heat Shock Proteins^7–9^. However, gene expression is regulated at multiple steps after RNA synthesis, such as translation and mRNA degradation. Post-transcriptional regulation allows cells to respond rapidly (*i.e.* seconds to minutes) to environmental changes. Post-transcriptional control of gene expression has been shown to regulate key virulence processes in *C. albicans*, such as the yeast-to-hyphae transition^10,11^, biofilm formation, stress responses^12^, evasion of innate immune responses^13^), and plays a role in cell wall synthesis^14^, a target of echinocandins. However, less is known about the contribution of post-transcriptional regulation to fungal cell membrane homeostasis during resistance.

A powerful technique for studying translation dynamics is ribosome profiling (ribo-seq)^15^, permitting global measurements of translation efficiency, ribosome occupancy and codon usage. However, in some contexts, ribosome profiling can be technically taxing. Previously, we have shown in budding yeast (*S. cerevisiae*) that ribosome dynamics and mRNA degradation are interconnected to the extent that 5’ to 3’ exonuclease degradation follows the last translating ribosome^16^. Thus, profiling 5’ monophosphorylated mRNA degradation intermediates can be used to provide information regarding ribosome dynamics reflecting rapid reprograming events in *S. cerevisiae*^17^. It is not known whether co-translational mRNA decay occurs in *C. albicans*. Establishing this approach in *C. albicans* would provide a valuable tool for investigating translation dynamics in isolation or in complex conditions^18^, such as in biofilms, within local host niches^19,20^ or inside macrophages. Understanding the transcriptional and translation responses in fluconazole-sensitive and resistant strains would be of high interest, especially at early time points where direct drug targets are more likely to be found. However, only one study^21^ has examined post-transcriptional responses to fluconazole in *C. albicans* using ribosome profiling. While Choudhary et. al. focus on translation responses after 6 h of fluconazole treatment in sensitive candida strains, no studies have yet investigated early post-transcriptional responses in sensitive and resistant strains.

Here, we have investigated the 5’P mRNA degradome in *C. albicans* and studied how in vivo ribosome protection patterns are altered in response to translation inhibitors and targeted depletion of amino acids. After demonstrating that 5PSeq provides a fast readout of in vivo ribosome dynamics in *Candida*, we investigated transcriptional and posttranscriptional responses to fluconazole treatment. We combined the use of RNA-Seq and 5PSeq, to study how sensitive (SC5314) and resistant (PLC124, MAY478 and MAY7) strains change their transcriptome at 30 min after fluconazole treatment (Table S1). Our work shows clear transcriptional differences between sensitive and resistant strains, even in the absence of drug treatment. Fluconazole addition leads to a clear upregulation of the ergosterol biosynthesis and other pathways. Contrary, despite a global change in ribosome dynamics across strains, the observed difference between strains were modest. In summary, our work provides insights into the early responses to fluconazole in sensitive and drug-resistant *C. albicans* and demonstrates the versatility of analyzing 5’P mRNA degradation intermediates to study ribosome dynamics.

## Results

### Co-translational mRNA decay in *C. albicans* enables the of study ribosome dynamics.

**Fig. 1:**
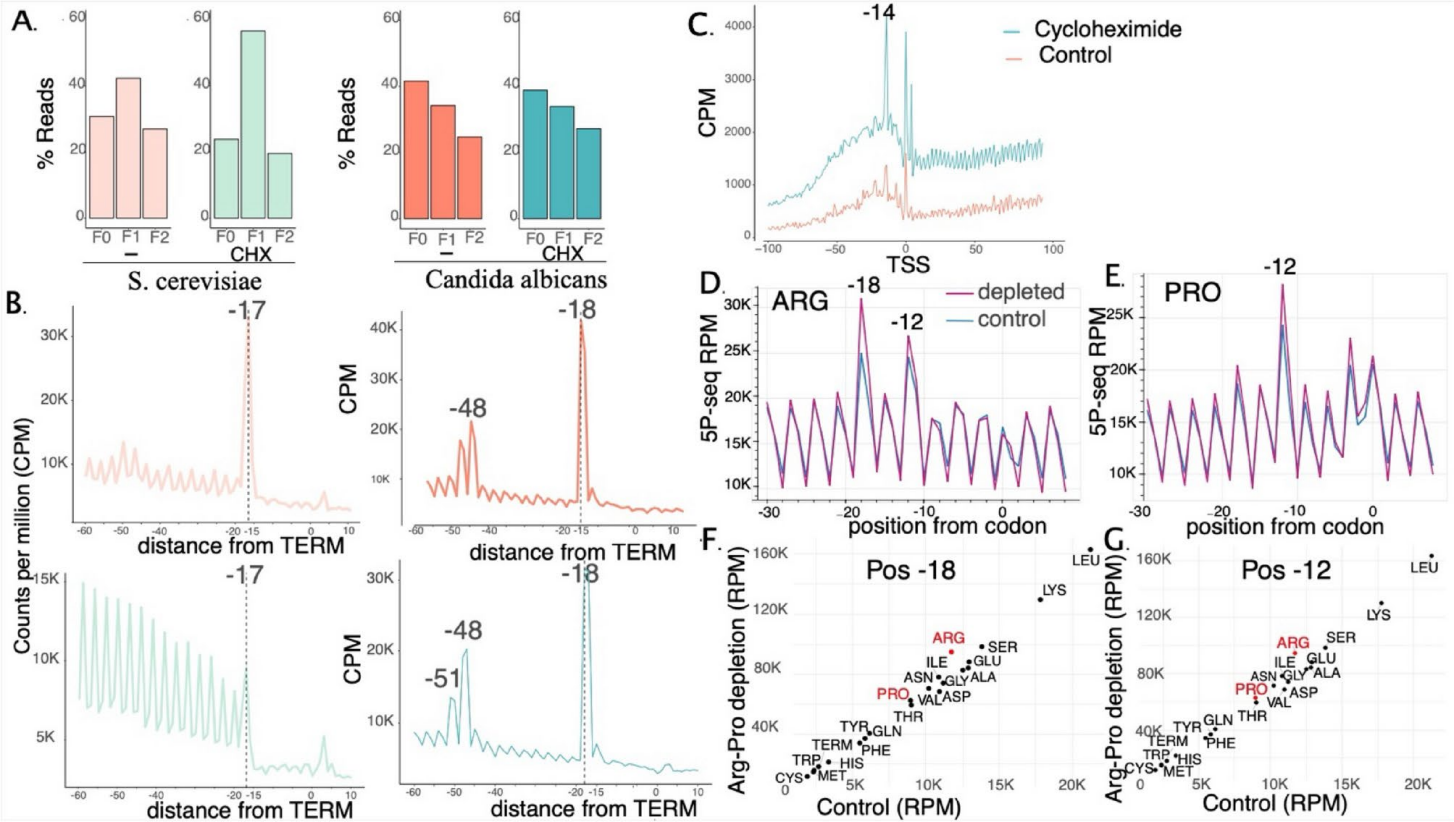
Co-translational decay in *C. albicans* enables the study of codon-specific ribosome pauses*.* (**A**) Barplot of 5Pseq read distribution across all translation frames genome-wide showing F0 is the preferred translating frame in *C. albicans,* whereas *S. cerevisae* preferentially uses F1. (**B**). Metagene of 5’P signal over 6031 genes in *C. albicans* with an accumulation of reads -18 nucleotides of the termination site (with a shoulder at -17) representing a ribosome protection 1 bp larger than the accumulated reads at -17 nt that predominate in the 6601 5’P signals in *S. cerevisae*. (**C**) Metagene plot of 5Pseq read counts normalized as Counts per Million (CPM) shows a ribosome stall 14 nt upstream of translation start upon treatment with cycloheximide. (**D**,**E**). Arginine and proline depletion show ribosome stalls at proline (position -12 from the codon) and arginine (position -12 and -18 from the codon). (**F**, **G**) A scatter plot of the 5PSeq read counts at stalled positions -18 and -12 of ARG, PRO depleted (y-axis) compared to control (x-axis) show stalls are specific to the depleted amino acids experimentally.

We have previously shown that sequencing the 5’P mRNA degradation intermediates in *S. cerevisiae* enables the study of in vivo ribosome dynamics^16^. Although related to *S. cerevisiae*, *C. albicans* is an ascomycetes yeast of the CTG clade, that predominantly translates the CTG codon as a serine rather than leucine^22^. Despite being separated from *S. cerevisiae* by 300 million years of evolution^23^, we hypothesized that co-translational mRNA decay might also enable the study of ribosome dynamics in *C. albicans*. To test this hypothesis, we performed 5’P sequencing in the laboratory strain SC5314 using HT-5PSeq^17^ (Table S2). As expected, 5’P signal showed the characteristic 3-nt periodicity associated with the 5’-3’ co-translational decay of ribosome protected mRNAs (Fig. 1A). However, contrary to what we have previously reported in *S. cerevisiae*^16^, the protection pattern was not centered in F1, and F0 protection was higher than F1 (Fig. 1A). This suggests that in *C. albicans* the ribosome protects a slightly longer fraction of the co-translational degraded mRNA. This can also be observed when investigating the ribosome protection pattern associated with the stop codon (Fig. 1B). In addition to the sharp protection at -17 nt characteristic of ribosomes paused at the stop codon in *S. cerevisiae*^16^, we also observed double protection peaks at -48 and -51 nt (with shoulders at -47/-50 nt). To better understand the protection offered by the ribosome during co-translational mRNA degradation, we perturbed translation elongation with cycloheximide (0.067 mg/mL for 15 min). Addition of cycloheximide is expected to stall elongating ribosomes and increase the observed 3-nt pattern^16^. Examining the 5’P read signal across all Open Reading Frames (ORFs) revealed a sharp accumulation of reads 14 nt upstream of the start site (Fig. 1C) and an increase in 3-nt periodicity (Fig. 1A), similar to what we have previously observed in *S. cerevisiae*^24^. We also observed that the sharp read accumulation 17/18 nt upstream of the termination site reflecting a slow terminating ribosome is maintained after cycloheximide treatment. The second double peak at -47 and -50 was also maintained, consistent with the protection of a collided ribosome stalled in close proximity to the ribosome located at the termination codon^24^. Interestingly, despite that cycloheximide addition led to a sharp termination associated protection at -17 nt, this protection retained a clear shoulder at -18 nt. This confirms that in *C. albicans* ribosomes provide a slightly longer in vivo protection during co-translational mRNA decay even in presence of cycloheximide. Thus, the increased protection in *C. albicans* is not exclusively caused by dynamic differences in ribosome conformation, as cycloheximide is known to stabilize a preferential conformation^25^.

To validate the ability of 5PSeq to measure ribosome stalls at codon resolution, we experimentally depleted selected amino acids to induce codon-specific ribosome stalls prior to 5PSeq analysis. We grew SC5314 for one hour in media depleted of proline, arginine and ornithine. The read density signal at the proline (cumulative signal over codons CCC, CCA, CCG) revealed a clear stall at position -12, coinciding with the entrance of the proline to the ribosome exit tunnel (Fig. 1E) similar to what we have previously shown in *S. cerevisiae*^26^. Looking at the signal of all codons at positions -12 (Fig. 1G) and -18 (Fig. 1F), we found that arginine and proline were clearly enriched suggesting the stalls were codon specific. Furthermore, we also observed a ribosomal pause at position -18 (with a shoulder at -17) at arginine (signal over codons CGC, CGA, AGA, AGG) corresponding to the arginine tRNA being positioned at the ribosome A site (Fig. 1D, F). This agreed with our expectations, since arginine limitation should lead to more ribosome stalling at that position. Reassuringly, also at codon level in vivo stalls displayed a double protection peak at F0 and F1 (-18/-17), as previously discussed. Taken together, we concluded that 5’P mRNA degradation sequencing enables the study of ribosome dynamics at single-nucleotide resolution in *C. albicans*.

## Early transcriptional and translational dynamic responses to fluconazole in sensitive *C. albicans*

**Fig. 2:**
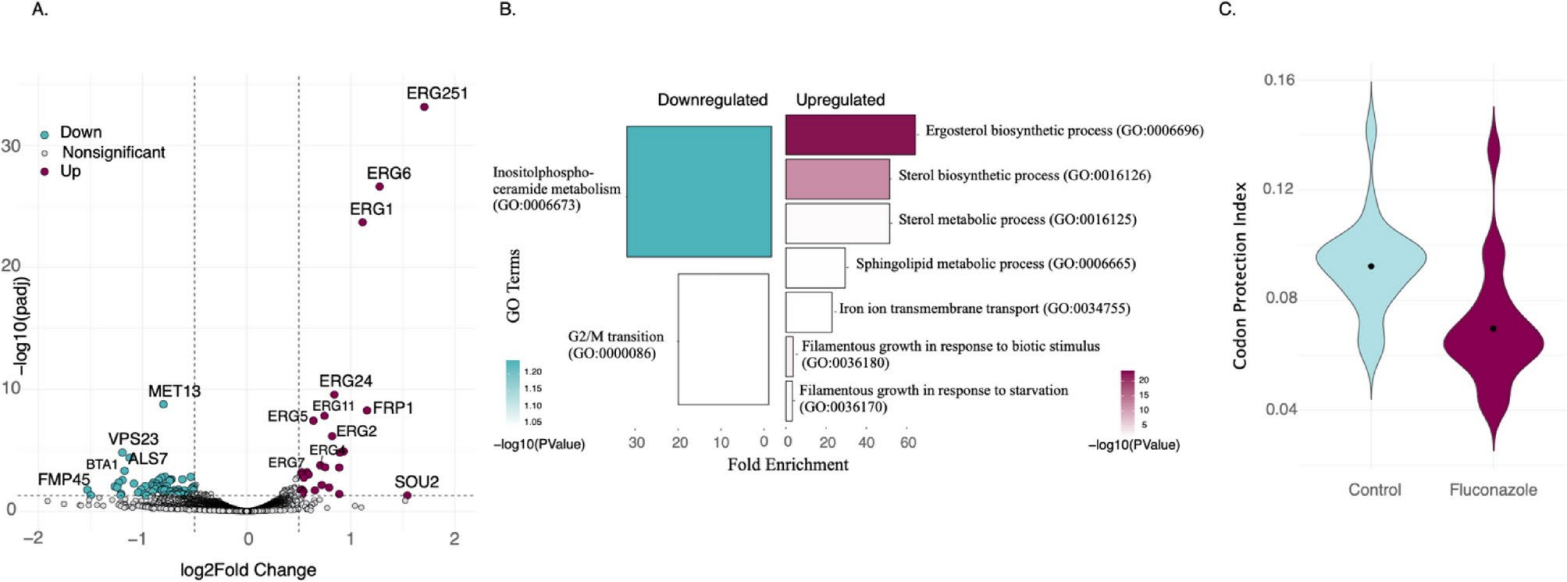
Early transcriptional response to fluconazole in sensitive *C. albicans*. (**A**) Volcano plot of the RNA sequencing data, corresponding to 6031 genes, expressed as log fold change Fluconazole/Control along the x axis and adjusted p value on the y axis. In plum: 53 genes upregulated after fluconazole in the ergosterol biosynthesis pathway. In blue: 78 genes downregulated genes in the inositolphosphoceramide metabolic process. The horizontal line shows an adjusted P value < 0.05. (**B**) Bar plot of fold enrichment of gene ontology terms for differentially expressed genes classified as upregulated (log2 fold change > 0.5) and downregulated in response to treatment with Fluconazole colored by adjusted P value. (**C**) Violin plots of codon protection index calculated as log_2_ (2F_i_/(F_total_-F_i_))), where F_i_ represents the number of 5’P read counts in the preferred Frame (F0), and F_total_ represents all frames for fluconazole treated (green, n = 3 biological replicates) and control (blue, n = 3 biological replicates). P-value = 0.0004476 using t-test.

Having established that 5PSeq can be used to study ribosome dynamics in *C. albicans*, we aimed to obtain a comprehensive understanding of the early transcriptional and post-transcriptional responses after fluconazole treatment. First, we investigated gene expression rewiring after 30 min treatment with 1 μg/ml fluconazole in sensitive *C. albicans* strain (SC5314) using RNA-Seq. We found 131 differentially expressed genes (FDR < 0.05), of which 74 genes were downregulated and 53 genes were upregulated (Fig. 2A, Table S3). Our results expanded prior work^4^ identifying 94 novel genes. Among the upregulated genes, we identified *ERG11* which encodes lanosterol 14-α-demethylase, the direct target of fluconazole. In fact, 28% of genes upregulated (n = 15) in response to fluconazole belonged to the ergosterol biosynthesis pathway (GO: 0006696). Two of these genes, *ERG1* and *ERG7* were upstream of the fluconazole target step (*i.e. ERG11*). Interestingly, *ERG3* was also found to be highly expressed, as well as the Major Facilitator Superfamily (MFS) drug efflux pump gene *Mdr1.* More genes were downregulated (n = 78, 1.2%) than upregulated (n = 53, 0.8%). Gene ontology analysis (Table S4) of these repressed genes revealed an enrichment in the inositol phosphoceramide metabolic process (GO:0006673) and G2/M transition of mitotic cell cycle (GO:0000086) (Fig. 2B), in agreement with previous work^4^. However, additional work will be necessary to confirm that those changes lead to differential protein accumulation.

Although fluconazole does not directly interfere with the translation process, we hypothesized that drug-induced cellular stress would perturb translational dynamics globally. Recently, Choudhary *et. al.*^21^ using ribosome profiling found that ROA1 PDR-subfamily ABC transporter genes, cell wall/cell membrane synthesis (*BMT7, OPI1, PGA1, ECM15, PEX11*) and stress responses (*PLC1*, *TRX1, GPI14*) genes were specifically upregulated at the translational level but not at the transcriptional level. Specifically, they treated the SC5314 strain with 1 μg/ml fluconazole for 6 h. However, we reasoned that at 6 h, cells could be adapted to the new condition and most of the translation responses would be associated with long-term fluconazole-induced stress/toxicity. By contrast, studying ribosome dynamics at a shorter time point could reveal particular mRNAs that are rapidly and transiently regulated in response to fluconazole.

To study changes in ribosome dynamics prior to long-term adaptation to fluconazole, we reasoned that 30 min treatment with fluconazole (1 μg/ml) would enable the study of both RNA-Seq and 5PSeq. Although 5PSeq cannot determine the fraction of mRNA associated to ribosomes, it can inform regarding codon specific ribosome stalls. Looking at the frame preferences of all codons (difference between peaks and valleys, F0/(F1 + F2)), we found a global decrease of 3-nt periodicity in response to fluconazole (Fig. 2C). However, we did not observe clear codon or gene specific differences. This suggests that at short times, even if there is a drastic change in ribosome dynamics, this effect is general and does not seem to affect particular genes.

## Transcriptional differences between resistant and sensitive *C. albicans*

**Fig. 3:**
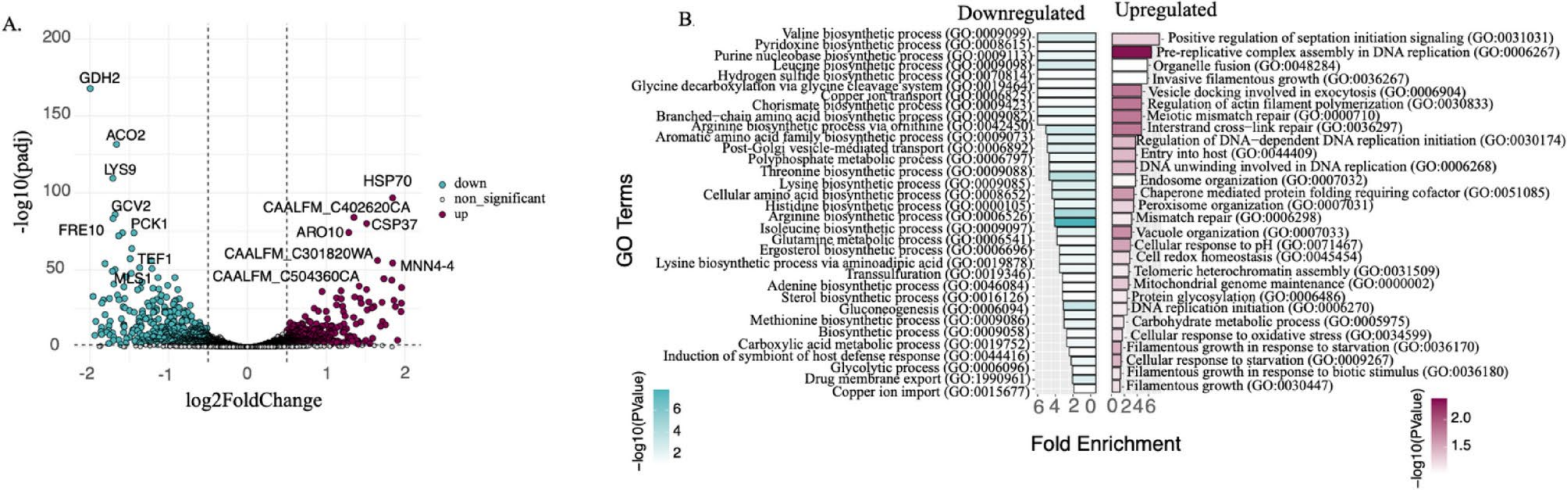
Transcriptional differences between sensitive and resistant *C. albicans* (**A**) Volcano plot of 6001 genes from transcriptome profiling by RNA-seq, seen as log fold change PLC124(resistant)/SC5314(sensitive) along the x axis and adjusted p value on the y axis. 852 genes upregulated (in plum) and 894 genes downregulated (blue). The horizontal line shows an adjusted P value < 0.05. (**B**) Bar plot of gene ontology terms (y-axis) plotted by fold enrichment (x-axis) and colored by adjusted P value.

Next, we characterized the transcriptome of fluconazole-resistant *C. albicans* strain (PLC124)^27^ and investigated the differences with respect to the sensitive SC5314. First, we characterized both strains in the absence of drug treatment. Whole transcriptome RNA-Seq analysis of the resistant clinical isolate PLC124 revealed substantial differences (29% of genes) compared to sensitive SC5314. Out of 6001 genes, we identified 1746 genes that were differentially expressed (FDR < 0.05), roughly equal proportions of upregulated (n = 852, 49%) and down-regulated genes (n = 894, 51%). Importantly, among the significantly upregulated genes we identified *Hsp70* (Fig. 3A)*,* a known stress response modulator involved in resistance.

Gene ontology analysis of the upregulated genes (Fig. 3B and Table S4) revealed an enrichment in the pre-replicative complex assembly involved in nuclear cell cycle DNA replication (GO: 0006267), vesicle docking involved in exocytosis (GO:0006904), regulation of actin filament polymerization (GO:0030833), meiotic mismatch repair (GO:0000710), and interstrand cross − link repair (GO:0036297). Whereas gene ontology enrichment revealed a prominent down-regulation of genes involved in ergosterol biosynthetic process (GO:0006696). Among the significantly downregulated genes, we identified *GDH2*, which encodes the NAD^+^-dependent glutamate dehydrogenase, which is a key enzyme responsible for amino acid-dependent ammonia production leading to extracellular alkalization^13^.

## Early responses to fluconazole in resistant *C. albicans*

**Fig. 4:**
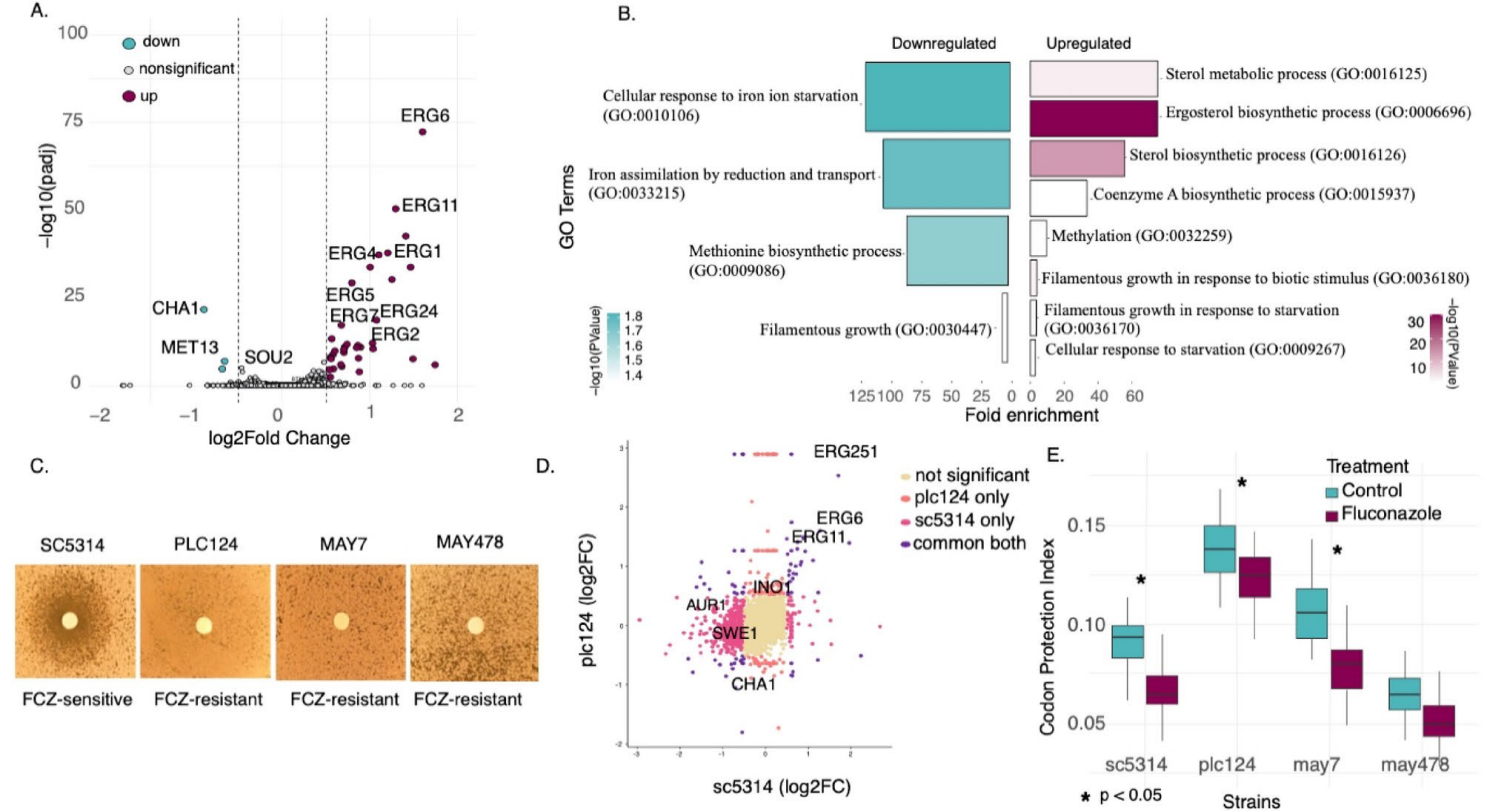
Transcriptional and post-transcriptional response after fluconazole in resistant *C. albicans* (**A**) Volcano plot of the whole transcriptome RNAseq data in PLC124, corresponding to 6006 genes, expressed as log fold change Fluconazole/Control along the x axis and adjusted p value on the y axis. In plum: 124 genes upregulated after fluconazole in the ergosterol biosynthesis pathway. In blue: 70 downregulated genes. The horizontal line shows an adjusted P value < 0.05. (**B**) Bar plot of fold enrichment of gene ontology terms for differentially expressed genes classified as upregulated (Fold change > 0.5) and downregulated in response to treatment with Fluconazole colored by adjusted P value. (**C**) Disk diffusion assay of *Candida* strains SC5314, PLC124, MAY7 and MAY478 ^27^; growth in presence of a disc with 25 μg fluconazole show resistance phenotype. (**D**). Comparison between SC5314 and PLC124 strains at transcriptional level; X-axis is log2FC of SC5314, and y-axis is log2FC of PLC124. (**E**) Box plots of codon protection index calculated as log_2_ (2F_i_/(F_total_-F_i_))), where F_i_ represents the number of 5’P read counts in the preferred Frame (F0), and F_total_ represents all frames for fluconazole treated (blue, n = 3 biological replicates) and control (purple, n = 3 biological replicates). P values obtained using t-test. Significant differences highlighted by *.

Having explored transcriptional differences between resistant and sensitive cells (Table S1) in absence of drug treatment, we investigated if the resistant strain responded differently to fluconazole treatment. RNA-Seq analysis in the resistant PLC124 isolate after 30 min treatment identified 194 differentially expressed genes (3%) (Fig. 4A). More genes were upregulated (n = 124, 64%) than downregulated (n = 70, 36%). Similar to the sensitive strain, the majority of genes in the upregulated group were uniformly enriched in the ergosterol (GO:0006696) and sterol (GO: 0016126) biosynthesis pathway (Fig. 4B). Of the downregulated genes, many were related to cellular response to iron starvation (GO:0010106), iron assimilation by reduction and transport (GO:0033215), methionine biosynthetic process (GO:0009086) and filamentous growth (GO:0030447) (Fig. 4B).

Next, we determined how different or similar the transcriptional responses were between SC5314 (sensitive) and PLC124 (resistant) strains (Fig. 4D). The two strains shared 72 genes in common, including *ERG6*, *ERG11* and *ERG251*. In contrast, 571 genes were unique to SC5314 including *AUR1* (downregulated), *SRR1* (downregulated) and *SWE1* (downregulated). Of the 128 genes unique to PLC124 were genes enriched in the biosynthesis of secondary metabolites including *CHA1* (downregulated, biosynthesis of aromatic amino acids), *ERG10* (upregulated, ergosterol biosynthesis) and *INO1* (upregulated, inositol biosynthesis). This suggests contribution to different aspects of yeast physiology, including amino acid biosynthesis, sterol biosynthesis, membrane biogenesis, and stress responses that are inherently distinct in resistant strains.

Finally, we explored how early ribosome dynamics responses differ across different strains (Fig. 4E). In addition to the sensitive SC5314 and the resistant PLC124, we also investigated MAY478, an additional clinical isolate^27,28^. MAY478 is aneuploid for chromosomes 4 and 6, previously linked to fluconazole resistance^28^. Despite confirming resistance by disk diffusion assay (Fig. 4C), we found a general decrease in frame protection (Fig. 4E). A clear decrease in frame protection can be seen for SC5314 and PLC124, while the difference was not significant for MAY478. However, the fact that a general decrease in frame protection could be observed for both sensitive and resistant strains suggests that early ribosome dynamics responses are not sufficient to discriminate between drug sensitive and resistant strains, contrary to what we have previously shown for ribosome targeting antibiotics in bacteria^18^.

## Discussion

In this study, we have investigated co-translational mRNA decay and early transcriptional responses to fluconazole treatment in sensitive and resistant *C. albicans*. We have sequenced the 5’P mRNA degradome using HT-5PSeq^17^. Our results show that, similar to *S. cerevisiae*, *C. albicans* displays a clear 3-nt periodicity for the 5’P mRNA degradation intermediates. However, in *Candida* the distance between the ribosome A site and the in vivo 5P’ boundary is 18 nt, instead of the sharp 17 nt observed in *S. cerevisiae*^16^. This is likely caused by an increased distance between the ribosome A site and the active site of the 5’-3' exonuclease co-translationally degrading the mRNA. We have confirmed that ribosome position is causative in the observed 5’P pattern by inhibiting translation elongation using cycloheximide and by performing a selective amino acid depletion. The selective depletion of proline and arginine led to codon-specific ribosome stalls at the depleted amino acids. To create a stronger limitation in proline availability and avoid a metabolic rewiring of *Candida* metabolism^29^, we also depleted arginine and ornithine, since arginine and ornithine are readily catabolized to proline. In addition to confirming the causative role of ribosome position in the 5’P mRNA degradation, the study of ribosome dynamics in *Candida* after proline depletion was of particular interest considering our recent work showing that proline catabolism is a key factor in *C. albicans* pathogenicity^27^.

After demonstrating that 5PSeq can provide a fast readout of ribosome dynamics in *C. albicans*, we investigated the early^30^ transcriptional and posttranscriptional responses to fluconazole in sensitive and resistant *C. albicans* strains using RNA-Seq and 5PSeq. Whole transcriptome changes in SC5314 occur as early as 15 min^4^ (0.83% of transcriptome, n = 48 DEGs). At 40 min of fluconazole treatment, more pronounced transcriptome changes are seen (2.86% of transcriptome, n = 165 DEGs). Here we expand on previous work by examining the transcriptome changes at 30 min, and find that 75 (1.2%) genes are upregulated while 154 genes (2.6%) are downregulated. Fluconazole addition leads to a clear upregulation of the ergosterol biosynthesis and sterol biosynthetic pathways in the sensitive SC5314 strain.

Furthermore, we established that the resistant *C. albicans* strain PLC124 exhibited inherent features of resistance, such as upregulation of Hsp70 and downregulation of *GDH2,* in the absence of fluconazole exposure. While Heat shock protein 90 (Hsp90) is known to confer fluconazole resistance in *C. albicans*^7–9^, less is known about Hsp70. In budding yeast Hsp70 family member Msi3 has been shown to confer tolerance to fluconazole^31^. Although striking, the apparent downregulation of Glutamate Dehydrogenase 2 *(GDH2*), remains an enigma. Gdh2 is one of the central enzymes in nitrogen metabolism, however, we have recently found that it is dispensable for escape from macrophages and virulence^13^.

Lastly, we characterize the transcriptional response to fluconazole in resistant *C. albicans* and show that it is similar to sensitive *C. albicans* strains, *i.e.*, upregulation of ergosterol biosynthesis genes (e.g., *ERG2, ERG4, ERG5, ERG6, ERG11* and *ERG24*) and downregulation of *MET13* and *CHA1*. These fluconazole-induced changes likely reflect disruptions in key metabolic pathways, impacting cellular growth, metabolism, stress response and secondary metabolite production. Next, we investigated the differences in early responses by comparing sensitive (SC5314) and resistant (PLC124) strains. Common mechanisms of acquired fluconazole resistance include drug target alteration (*i.e.,* missense mutations in *ERG11*), overexpression (*e.g.,* aneuploidy), and cellular stress response pathways that enable survival in drug-induced stress states. Two critical stress response pathways that mediate azole resistance involve the Heat Shock Proteins and *ERG3* (39). Hsp90 is a molecular chaperone that stabilizes folded proteins acting as signal transducers in the PKC-MAPK cell wall integrity pathway (Pkc1, Bck1, Mkk2, Mkc1) of the *C. albicans* stress response^8,30^. Loss of function mutations in the *ERG3* (Δ-5,6-desaturase) gene lead to accumulation of the toxic sterol 14-α-methyl-3,6-diol byproduct of the ergosterol pathway, which would otherwise accumulate in response to fluconazole. Hsp90 and Pkc1 are required for *ERG3*-dependent azole resistance^5^. Consistent with this, we observed that that PLC124 strain displayed upregulated genes involved in virulence and drug efflux such as the chaperone Hsp70 and the drug efflux pump Pkc1*.*

Previous work using ribosome profiling and longer time (6 h) of fluconazole exposure (1 μg/ml) reported subtle translational differences in transporter genes (ROA1), genes involved in cell wall and membrane synthesis (*BMT7, OPI1, PGA1, ECM15, PEX11*), and stress response genes (*PLC1, TRX1, GPI14*). These genes were specifically upregulated at the translational level, but not at the transcriptional level^21^. Using 5PSeq at shorter times (30 min) we observed only modest changes in ribosome dynamics. This suggests that the previously reported translational changes could be associated with the long-term fluconazole toxicity. Additionally, it is important to consider that ribosome profiling and 5PSeq query different subsets of ribosomes in the cell^17^. Ribosome profiling investigates the bulk of the ribosomes in the cell, while 5PSeq focuses on those ribosomes protecting mRNA undergoing co-translational degradation. Despite this, we observed that fluconazole treatment led to a generalized decrease of the observed 3-nt periodicity. As this affects both fluconazole resistant and sensitive strains, we hypothesize that it is the result of the stress associated with rewiring of the translation process after fluconazole treatment and does not inform regarding the long-term ability of the strains to grow in the presence of fluconazole. This is consistent with the fact that fluconazole does not directly interfere with the translation process.

In summary, our work provides additional details regarding the transcriptional rewiring of sensitive and resistant strains to fluconazole treatment. Additionally, we demonstrate that 5PSeq can be used in *C. albicans* to obtain single-nucleotide information regarding in vivo ribosome dynamics. We envision that 5PSeq will be used in the future to study multiple stress conditions in *C. albicans*, including oxidative stress. Importantly, we have recently shown that 5PSeq can be used to investigate complex microbial samples even in the clinical context without the technical hurdles associated with other approaches such as ribosome profiling^15^. As amino acid metabolism is a key factor to understand *C. albicans* virulence^27,29^ we envision that the future application of simplified 5PSeq to complex samples would facilitate the study of amino acid metabolism during fungal infection.

## Materials and methods

### *Candidaalbicans* strains and culture conditions

*C. albicans* reference strain SC5314 and fluconazole resistant clinical isolates PLC124, MAY7 and MAY478 (Supplementary Table 1) were maintained on YPD (yeast extract 1%, peptone 2%, and glucose 2%). To confirm the fluconazole resistance phenotype, a disk diffusion assay was performed. Briefly, cells from overnight YPD cultures were subcultured in fresh YPD medium at OD_600_ = 0.2 and grown for 3 h at 30 °C to an OD_600_ = 1.5–2.2. These log-phase cultures were diluted in 10 ml of YPD at ~ 4 × 10^4^ cells/ml and 3 ml aliquot were overlayed on dry YPD plates and the liquid allowed to be completely absorbed. Sterile disks pre-impregnated with fluconazole (25 μg) or 2% DMSO in H_2_O for control were aseptically placed on top of the agar. The plates were incubated at 35 °C for 24 h and photographed; the assays were repeated 3 independent times.

Fluconazole treatment: cultures were pre-grown overnight at 30 °C with constant shaking. The following day cultures were diluted to OD_600_ of 0.1. The starting culture was split into two tubes (2 mL per tube) and subsequently grown at 30 °C aerated until an OD_600_ of 1.5 – 2.2. One tube was treated with 2% DMSO (diluent control), while the second tube was treated for 30 min with 1 μg/ml fluconazole (2 × MIC, experimentally determined for the SC5314 strain used in this study^27^). Cells from three biological replicates were harvested by centrifugation and flash frozen in liquid nitrogen.

Cycloheximide treatment: cultures were pre-grown as for fluconazole treatment. The following day cultures were used to inoculate two flasks (150 ml per flask) at an OD_600_ of 0.1. When the cultures reached an OD = 1, one culture was treated for 15 min with 1 mL cycloheximide working solution in ethanol (final 0.067 mg/ml concentration in media), whereas the control culture received 1 mL of 90% Ethanol. Cells from three biological replicates were harvested by centrifugation and flash frozen in liquid nitrogen.

Amino acid deprivation: strain SC5314 was pre-grown overnight in 3 mL YPD shaking at 30 °C. Cells were harvested by centrifugation and resuspended at an OD_600_ of 0.2 in proline starvation media. Proline starvation media is YNB (Yeast Nitrogen Base without amino acids and ammonium sulfate; 0.17%) supplemented with glucose (final concentration 2%) and all amino acids (0.2 g/L) except Proline, Arginine and Ornithine. Cells from three biological replicates were harvested by centrifugation and flash frozen in liquid nitrogen.

### RNA extraction and HT-5Pseq library preparation

Total RNA was extracted from frozen cell pellets using the RiboPure-Yeast RNA Isolation Kit (Thermo Fisher) and RNA concentrations were measured using a Nanodrop 2000c Spectrophotometer (Thermo Fisher Scientific). HT-5’P sequencing library preparation was performed as described in Zhang et. al.^17^. Briefly, we used 6 μg of total RNA as input. Samples were subjected to DNAse treatment using TURBO DNAse enzyme for 20 min followed by purification with 2.5X volume of absolute ethanol and 1/10 volume NaAc 3M and overnight precipitation at -20 °C. After resuspension, DNA-free RNA was subjected to single stranded RNA ligation with 100 μM rP5_RND oligo (final 10 μM) for 2 h at 25 °C with 10 Units of T4 RNA ligase 1 (NEB). Ligated RNA was purified using 2 volumes RNA Clean XP (Beckman Coulter) as per manufacturer’s instructions. Total ligated RNA was reverse transcribed with Superscript II (Life Technologies) and primed with Illumina PE2 compatible oligos containing random hexamers (final 1 μM) and oligo-dT (0.5 nM). The reaction was carried out in the Thermocycler for 10 min at 25 °C, 50 min at 42 °C and heat inactivated for 15 min at 70 °C. After reverse transcription, the RNA in the cDNA/RNA mixture was depleted by incubation 20 min with sodium hydroxide (40 mM) at 65 °C followed by Tris–HCl, pH = 7.0 (40 mM) neutralization. After reverse transcription and DSN reactions, a cleanup step was carried-out using Ampure XP beads (Beckman Coulter) as described previously^17^. For the fluconazole and cycloheximide experiments, ribosomal RNA was depleted using a DSN (Duplex specific nuclease) reaction and a mixture of probes targeting the 18S rDNA, 25S rDNA and 5.8S rDNA targeting partial ribosomal RNA regions as in^26^. The reaction was denatured at 98 °C for 2 min and incubated at 68 °C for 5 min before adding a prewarmed DSN buffer mix with 1 Unit of DSN enzyme (Evrogen) and carried out for 20 min at 68 °C. To stop the reaction, we added a 2X DSN stop solution for 10 min at 68 °C. The resulting library was PCR amplified using 2X Phusion High-Fidelity PCR Master Mix with HF Buffer (NEB) and 0.25 μM of PE1.0 and multiplex PE2.0_MPX. The Thermocycler program was 30s at 98 °C, 15 cycles x (20s at 98 °C, 30s at 65 °C, 30s at 72 °C) and 7 min at 72 °C. HT-5’P sequencing libraries were size selected to 300–600 bp with an average library size of 450 bp using AMPure XP beads (Beckman Coulter) 0.7x-0.9 × Vol. and sequenced on Illumina NextSeq 2000 platform with a depth of 20 M reads on average.

### RNAseq library preparation

Ribosomal RNA was depleted from ~ 1 µg of total RNA using riboPOOLs (siTOOLs Biotech) kit as recommended by the manufacturer. After depletion samples were precipitated using 2.5 volumes of 95% (vol/vol) ethanol, a 1/10 volume of 3 M sodium acetate and 1 µL of glycoblue, mixed and incubated overnight at − 20 °C. Concentration was done by centrifugation at 14,000 × g, 30 min at 4 °C, the pellets were washed twice with 70% ethanol, dried and resuspended. For library preparation, we used the NEBNext Ultra directional RNA Library Prep kit for Illumina (New England BioLabs) as recommended by the manufacturer for relatively low input,13 amplification cycles, and mean library size was around 350 bp. Libraries were sequenced on Illumina NextSeq 2000 platform with a depth of 20 M reads per sample.

### Read processing and quantification

Base calls (BCL) were converted to fastq files and subsequently demultiplexed using the Illumina bcl2fastq v2.20.0.422 tool with option –barcode-mismatches 1. Sequencing adapter read trimming at 3’ end was applied using cutadapt V3.1 with Python 3.7.2 (https://cutadapt.readthedocs.io/en/v3.1/installation.html). The 5’ end 8-nt random barcodes were extracted and added as UMI to the header of fastq files using UMI-tools^32^. Reads were mapped to the SC5314 reference genome (ASM18296v3) for *Candida albicans* using STAR version 2.7.9a^33^ with parameter –alignEndsType Extend5pOfRead1 to exclude 5’end soft-clipped bases. In addition, reads were mapped to mRNA, rRNA, tRNA, snRNA, snoRNA and ncRNA separately with corresponding indexes generated by STAR 2.7.9a to calculate RNA content composition (see Supplementary Table 1). Duplicated 5’ read ends introduced by PCR during library preparation were collapsed and removed based on random barcode sequences with UMI-tools (31). Low complexity samples (may478_fluc_rep01, may478_ut_rep01, plc124_ut_rep01) were removed from further analyses (see Supplementary Table 2). To compare differences in 5P’ read coverage at gene level, reads per CDS were counted using Subread package (featureCounts) using the options -t CDS -g ID -s 0 -T 8^34^**.** Differential gene expression analysis was performed using DeSeq2^35^. The threshold for differentially expressed genes was defined as p-value < 0.05 and log2FoldChange > 0.5. We adjusted the alpha parameter to 0.5, corresponding to FDR threshold 50% to increase the number of Differentially Expressed Genes (DEG) to 61. Analysis of 5’ ends positions was done using a modified version of the Fivepseq package^36^ that accounts for the CUG codon reassignment from Leucine to Serine in *C. albicans*^22^. Briefly, the 5’ mRNA reads are summed in biological samples and normalized to reads per million (rpm). The relative position of 5’ reads with respect to all codons of all open reading frames (ORF) were summed at each position. Metagene plots are generated by plotting the summed reads within a window of each annotated start and termination site, or codon. The amino acid and codon frame protection index was calculated as log_2_(2F_i_ /(F_total_- F_i_)), where F_i_ where is the preferred frame, and F_total_ is the sum of counts across all three translation frames. Functional annotation analysis was done using the DAVID Bioinformatics Resources^37^. Figures were generated using ggplot2^38^ in R studio Version 2022.12.0 + 353.

### Transcript identification and differential expression analysis

Reads from RNAseq were mapped to the SC5314 reference genome (ASM18296v3) for *C. albicans* using STAR version 2.7.9a^33^. The resulting files were converted to binary format (bam), then sorted and indexed using samtools version 1.14. Transcript counting was done using the Subread featureCounts version 1.5.1 with the option ‘-t CDS’. Each strain was analyzed independently to identify differentially expressed genes (DEGs) using rlog normalization based on the negative binomial distribution using DeSeq2 version 1.38.3. All plots were generated in RStudio Version 2023.06.1 + 524. Identified up- and down-regulated genes with p-adjusted value < 0.05 were analyzed using the DAVID Bioinformatics Resources^37^ and the resulting enriched gene ontology terms were plotted in Rstudio.

### Statistical analysis

The statistical significance of the peaks observed in Fig. 1A were calculated assuming a Poisson distribution of meta-counts with lambda equal to the 0.84 quantile and probability of a count falling into the distribution being considered as the significance p-value for that count being a peak. Statistical tests include t-test using the t.test() function.

## Supplementary Information

Supplementary Information 1.

Supplementary Information 2.

Supplementary Information 3.

Supplementary Information 4.

Supplementary Information 5.

## Data Availability

The raw sequencing files were deposited in the GEO database under Accession No. GSE267940 and GSE267941. All scripts and count data used in the study are available at https://github.com/irenestevens8/Candida_degradome. A modified Fivepseq pipeline^12^ to account for Candida’s alternative nuclear code is available at: https://github.com/irenestevens8/fivepseq/tree/Candida.
