# Supplementary material for: The early transcriptional and post-transcriptional responses to fluconazole in sensitive and resistant Candida albicans
Source: Sci Rep. 2024 Nov 22;14:29012. doi: 10.1038/s41598-024-80435-w (PMC11586853; doi:10.1038/s41598-024-80435-w)
Supplement: Supplementary file 1 — Supplementary Information 1. [file 41598_2024_80435_MOESM1_ESM.docx]

# **Supplementary**

Table S1: *Candida albicans* strains used in study

Table S2: Sequencing and alignment quality metrics

Table S3: Differentially expressed genes

Table S4: Enriched DAVID Terms (related to Figures 2 and 4)
